# Sanicula

**Published:** 1890-08

**Authors:** Daniel W. Clausen


### SANICULA.—A CAUTION.

EDITOR HOMŒOPATHIC PHYSICIAN:

I wish it to be particularly understood that my "Caution to Physicians," in regard to Sanicula, which appeared in your June number, was in no wise intended as a reflection on Messrs. Bœricke & Tafel, for I have too much confidence in the integrity and the pharmaceutical ability of those gentlemen to believe that any preparation would be sent from their pharmacy but that which is *bona fide*. My "Caution to Physicians" was a caution against carelessness on *their* part, or neglect to ask for a remedy by its full and proper name.

One would scarcely have supposed that this explanation would become necessary, but my "caution" has been mistaken for a "reflection," and, in justice to Messrs. Bœricke & Tafel, I desire to remove a wrong impression from the minds of any who might entertain it.

Respectfully yours,

DANIEL W. CLAUSEN, M. D.

